# Vestibular Migraine: Clinical Challenges and Opportunities for Multidisciplinarity

**DOI:** 10.1155/2016/6179805

**Published:** 2016-12-19

**Authors:** Isabel Luzeiro, Leonel Luís, Freire Gonçalves, Isabel Pavão Martins

**Affiliations:** ^1^Neurology Department, Coimbra Hospital and University Centre, Coimbra, Portugal; ^2^Centre for Sleep Medicine, Coimbra Hospital and University Centre, Coimbra, Portugal; ^3^Laboratory for the Study of Language, Faculty of Medicine, University of Lisboa, Lisboa, Portugal; ^4^Otorhinolaryngology Department, Cascais Hospital, Cascais, Portugal; ^5^Faculty of Medicine, Coimbra University, Coimbra, Portugal

## Abstract

Migraine and vertigo are two very prevalent conditions in general population. The coexistence of both in the same subject is a significant clinical challenge, since it is not always possible to understand whether they are causally related or associated by chance, requiring different diagnostic and therapeutic approaches. In this review we analyze and summarize the actual knowledge about vestibular migraine (VM), focusing on the new concepts proposed by the International Classification of Headache Disorders 3-beta and by the Bárány Society and also addressing the former concepts, which are still present in clinical practice. We conclude that clinical studies using a multidisciplinary approach are crucial in this field, since different specialists observe the same pathology with different eyes. Clinical presentation of VM is variable in what concerns vestibular symptoms temporal relation with migraine headache, as well as in their accompanying manifestations. Biomarkers, either genomics or functional, and molecular imaging techniques will be helpful to clarify many aspects of the complexity of this entity, helping to define to what extent can VM be considered a separate and independent clinical entity.

## 1. Introduction

Complaints of vertigo or dizziness are common in patients with migraine [1] and can be reported by 30–50% of subjects, at least occasionally [1]. Recently, there was an increased interest on these issues due to the finding, in epidemiological studies, that the cooccurrence of those two conditions was higher than expected from the combined prevalence of both disorders in general population [2] and the estimation that vestibular symptoms associated to migraine can represent the most common cause of vertigo. Recently, vestibular migraine (VM) was integrated as an independent entity in the appendix of the International Classification of Headache Disorders 3-beta (ICHD-3 beta, A1.6.5) [3]. This designation and definition criteria resulted from a consensus document published by the International Headache Society (IHS) and the Bárány Society (International Society for Neurootology), being established by a collaboration between neurologists and otorhinolaryngologists (see “ICHD-3 Beta Diagnostic Criteria of Vestibular Migraine” below). Yet, the broad definition of this disorder has been criticized due to its lack of specificity [4, 5]. Vertigo of vestibular origin affects 7.4% of general population and migraine is more common, being present in 12–16% of the population [1, 2, 6]. If totally unrelated, the cooccurrence of both conditions in the same individual should be expected to occur in 1% of the population. In fact, this percentage is 3.2%, which corresponds to three times more, suggesting the association is more than just casual [2, 6, 7].

*ICHD-3 Beta Diagnostic Criteria of Vestibular Migraine*

- At least five episodes filling criteria (C) and (D)
- Current or past history of migraine, either with or without aura, considering the International Classification of Headache Disorders (ICHD) criteria1
- Moderate or severe vestibular symptoms^2^^,^ 3, lasting 5 minutes to 72 hours^4^
- At least half of the episodes are associated with at least one of these migrainous features^5^:
  1. Headache with at least two of the following characteristics:
    - Unilateral location
    - Pulsating quality
    - Moderate or severe intensity
    - Aggravation by routine physical activity
  2. Photophobia and phonophobia6
  3. Visual aura7
- No other better diagnostic explanation8

When occurring in the same individual, they can be associated or separated in time, frequently integrating migraine phenomenology and giving rise to the description of several (apparently) different conditions: VM, migraine with brainstem aura (MBA), or hemiplegic migraine (HM) [3, 8]. Clinical manifestations of these distinct entities seem to be part of a continuous spectrum where MBA and HM are in one end, followed by VM and migraine without aura in the other end. Migraine can also cooccur in the same patient with other vestibular disorders (such as Ménière's disease [9] and benign paroxysmal positional vertigo [VPPB] [9–11]). Moreover, anxiety may be the sole cause of dizziness in about 30% of cases [11], contributing to increase complexity in evaluating those complaints in clinical practice.

In this review, we will present an overview about the historic evolution of concepts leading to what is currently known as VM, we will focus on migraine clinical manifestations, stressing on the migraine subtypes that include vertigo in their clinical spectrum, and we will finally focus on VM and on its differential diagnosis, pathophysiology, and treatment.

## 2. Historical Perspective

Despite the well-known relationship between migraine and vertigo in children [12] the association between vestibular symptoms and migraine in adults was firstly recognized in 1984 [13, 14]. In 1961, Bickerstaff made the first description of migraine with brainstem symptoms, including vertigo, and called it basilar artery migraine [15] in two patients with identical symptoms, suggesting an abnormality in basilar artery circulation. He referred that the earliest recorded description of basilar artery migraine was made by Aretaeus of Cappadocia in 131 BC [15]. In 2004, the International Headache Society (IHS) has reclassified it as basilar migraine, considering the uncertainty of basilar artery involvement [16]. More recently, in the ICHD-3 beta classification (2013), it became known as migraine with brainstem aura (MBA) [3].

Boenheim, in 1999, used the term vestibular migraine (VM) for the first time. In 2001, Neuhauser et al. proposed the diagnostic criteria for Migrainous Vertigo, which have been widely used since then [17, 18]. More recently (2013), a working group (Bárány Society and International Headache Society) [3] established an expert consensus on the diagnostic criteria currently in use, which are included in an appendix of the third edition of the International Classification of Headache Disorders. However, clinical features of this entity are still under discussion and are still a matter of research.

## 3. Migraine Clinical Manifestations

Migraine is a common, recurrent chronic disease associated with acute episodes with two major subtypes: migraine with aura and migraine without aura. Both of them are characterized by headache episodes with a variety of accompanying symptoms, namely, 4–72 hours of headache duration, pulsating quality, moderate to severe pain intensity, usually unilateral location, and aggravation with routine physical activity like climbing stairs or walking or avoidance of these activities. During headache, the occurrence of nausea and/or vomiting is mandatory and photophobia and phonophobia are also present [3].

In migraine with aura, aura defining symptoms are multiple, are of different types (visual, sensory, motor, etc.), and usually follow a successive presentation during a variable period of time (from 5 min to one hour). Headache normally occurs in a period of 60 minutes after aura initiation.

According to ICHD-3 beta, we can consider three entities that comprise vertigo in the spectrum of possible clinical manifestations: MBA, sporadic and familial hemiplegic migraine (HM), and benign paroxysmal vertigo (BPV). MBA and HM are subtypes of migraine with aura. BPV is included in “episodic syndromes that may be associated with migraine” and occurs in healthy children. Between self-limited episodes, neurological examination, audiometry, vestibular functions, and electroencephalogram (EEG) must be normal. A unilateral throbbing headache may occur during attacks but is not a mandatory criteria [3]. The relationship between BPV and VM needs to be further deepened.

### 3.1. Migraine with Brainstem Aura (MBA)

MBA is considered by some authors as a subtype of a migraine with aura [19] and by others as more similar to HM [20]. It has this designation because headache is associated with brainstem or occipital lobes dysfunction. By definition, according to ICHD-3 beta (see “ICHD-3 Beta Diagnostic Criteria of Migraine with Brainstem Aura” below), the occurrence of a minimum of two episodes is required to make the diagnosis. In this type of migraine, there is no motor weakness. Aura should consist of visual, sensory, and/or speech/language symptoms and be fully reversible and at least two of brainstem symptoms (dysarthria, vertigo, tinnitus, hearing loss, diplopia, ataxia, or decreased level of consciousness) must be present. Furthermore, it is necessary that each aura symptom develops in more than 5 and less than 60 minutes and that at least one is unilateral and followed by headache. Headache is mainly bilateral, referred to cervical or occipital region [21], and begins in many cases alongside the aura [20]. Triggering factors are common to other entities with a slight predominance of sleep and emotional disorders [22]. More than one-third of the first attacks arise in the first decade of life and 2/3 in the second and third decades [19, 20, 22, 23] and it predominantly affects women [19, 23, 24].

*ICHD-3 Beta Diagnostic Criteria of Migraine with Brainstem Aura*

- Two or more attacks according to criteria (B)–(D)
- Aura manifested by visual, sensory and/or speech/language symptoms, each totally reversible, without motor or retinal symptoms
- Two or more of the following brainstem manifestations:
  1. Dysarthria
  2. Vertigo
  3. Tinnitus
  4. Hypoacusis
  5. Diplopia
  6. Ataxia
  7. Decreased level of consciousness
- Two or more of the following four features:
  1. One or more aura symptom spreads gradually over a minimum of 5 minutes, and/or two or more symptoms occur sequentially
  2. Each single aura symptom lasts from 5 to 60 minutes
  3. One or more aura symptom is unilateral
  4. Headache occurs simultaneously or within 60 minutes after the aura
- A transient ischaemic attack has been ruled out and there is no other ICHD-3 diagnosis that better explains the symptoms

MBA requires differential diagnosis with cerebrovascular diseases, seizures, and some genetic conditions such as cerebral autosomal dominant arteriopathy with subcortical infarcts and leukoencephalopathy (CADASIL), mitochondrial encephalomyopathy, lactic acidosis and stroke-like episodes (MELAS), and pathology of the posterior fossa.

Several studies recognize diplopia as the most frequent aura manifestation immediately followed by vertigo and tinnitus [22], while others point vertigo as the most common aura manifestation, occurring in 61–63% of patients [19]. The prevalence of vertigo and tinnitus, often as the first symptoms, raises the question, in pathophysiological terms, of a connection to VM. Since brainstem aura (namely, vertigo) in order to meet ICHD-3 beta criteria [3] must last from 5 to 60 minutes and be followed by headache [3], when this does not happen [25], VM should probably be considered as the most likely diagnosis [21, 26]. When vertigo is the aura and these criteria are met, migraine can be classified either as MBA or as VM.

There are no epidemiological studies regarding the prevalence of MBA [19]. In the retrospective study by Kirchmann et al. [19], the prevalence of migraine with brainstem signs in a group of patients with nonhemiplegic migraine with aura was 10%; in a more recent study carried out in China it comprised 6.6% [22]. In another study by Eriksen et al. [27], in patients with migraine with aura, the prevalence of MBA was 7%. It is estimated that 3–17% of children and adolescents with migraine have MBA.

### 3.2. Hemiplegic Migraine (HM)

Nearly 100 to 200 families with familial HM (FHM) have been identified and about 200 cases of sporadic HM have been reported. Clinically, patients have normally paresis in addition to other focal signs. FHM is an autosomal dominant type of migraine with aura. Diagnostic criteria require the presence of reversible motor deficits that are associated with at least 1 other transient neurological symptom and identical episodes in at least 1 first- or second-degree relative. The phenotype of FHM is variable including paroxysmal episodes with episodic ataxia, brainstem related aura, coma, seizures, and deafness [28]. All types may occur with vertigo or associated with episodic ataxia, especially FHM type I (associated with CACNA1A gene). Ataxia is the most consistent phenotype, present in 20% of cases, and may be the only clinical manifestation [28, 29]. Currently, we recognize three types of FHM, each of them linked to a different gene [8]: CACNA1A, ATP1A2, and SCN1A. The only prevalence study was conducted in Denmark and it showed a prevalence of 0.003%, but in Portugal there are many families identified either with HM (namely type I) or with episodic ataxia type 2 or spinocerebellar ataxia type 6 [28–31]. Diagnostic criteria are as follows.

*ICHD-3 Beta Diagnostic Criteria of Hemiplegic Migraine*

- Two or more attacks according to criteria (B) and (C)
- Aura manifested by both of the following:
  1. Motor weakness that is totally reversible
  2. Visual, sensory, and/or speech/language symptoms that are completely reversible
- Two or more of the following four features:
  1. One or more aura symptoms spreads gradually over a minimum of 5 minutes and/or two or more symptoms occur sequentially.
  2. Each single aura symptom lasts from 5 to 60 minutes and motor symptoms last less than 72 hours.
  3. One or more aura symptoms are unilateral.
  4. Headache occurs simultaneously or within 60 minutes after the aura.
- Transient ischaemic attack and stroke which have been ruled out and there is no other ICHD-3 diagnosis that better explains the symptoms

### 3.3. Benign Paroxysmal Vertigo (BPV)

BPV, one of those “episodic syndromes related to migraine,” previously named migraine equivalents (see “ICHD-3 Beta Diagnostic Criteria of Benign Paroxysmal Vertigo” below), is common in children [3]. With a prevalence of 2 to 2.6% and a frequent association with VM (21–80% of cases) [32, 33], BPV is considered the most common precursor of VM, although a later development of this entity is not mandatory [34, 35]. Both genders are equally affected by this condition [32]. BPV is characterized by recurrent attacks of vertigo (or its interpretation through the observation of child unsteadiness), appearing without warning and often associated with nystagmus, ataxia, vomiting, paleness, and fear, without loss of consciousness and resolving spontaneously after minutes to hours [3, 36]. More commonly, vertigo lasts less than 5 minutes [36] with headache occurring simultaneously or after it. The diagnosis requires five episodes of vertigo resolving spontaneously after minutes to hours and normal neurological and audiovestibular examination between the paroxysms.

*ICHD-3 Beta Diagnostic Criteria of Benign Paroxysmal Vertigo*

- Two or more attacks according to criteria (B) and (C)
- Vertigo appearing without prodromes, maximal at onset, with spontaneous and complete improvement within minutes to hours and without loss of consciousness
- One or more of the following additional symptoms or signs: (1) nystagmus, (2) ataxia, (3) vomiting, (4) pallor, and (5) fearfulness
- Normal neurological examination and audiometric and vestibular functions between attacks
- Not better explained by another disorder

### 3.4. Vestibular Migraine (VM)

#### 3.4.1. Diagnostic Criteria

In the previous IHS classification (ICHD-2, 2004) VM was not included and vertigo was rather considered a symptom of MBA (except in HM) [16]. Actually, VM was only added in the appendix of the ICHD-3 beta. Diversity of nomenclature and different diagnostic approaches, besides the intrinsic complexity of the diagnosis itself [37], led to the elaboration of consistent criteria, that could contribute to organize and homogenize VM clinical approach (see “ICHD-3 Beta Diagnostic Criteria of Vestibular Migraine”).

Although all criteria for VM diagnosis required episodic or recurrent vestibular symptoms [14] ICHD-3 beta diagnostic criteria require more episodes (5 episodes) and more severity (moderate to severe) when compared to Neuhauser et al. criteria [17] and further exclude probable vestibular migraine [3]. Therefore, this syndrome is conceptualized as a chronic recurrent disorder like migraine headache and should not be diagnosed in isolated episodes of vertigo in migraine sufferers. The diagnosis of VM requires the presence of headache, a throbbing headache, and dysautonomic signals and symptoms. Headache should be preceded or followed, immediately or after a variable period of time, by an attack of vertigo or dizziness [26]. However, the temporal relation between these two main symptoms is not fixed. Vertigo can arise in the context of a headache attack (preceding, occurring with, or after) [17] or between attacks, and the temporal relationship is extremely varied [3]. Vertigo episodes have a variable duration from clusters of seconds (10%), occurring repeatedly for a period of time, to attacks during minutes (3%) [38], days (30%), and rarely weeks [2, 39]. By definition, they last from minutes to hours or days (usually, less than 72 h), with the sum of the clusters being considered as one attack. Vertigo must be moderate to severe, interfering with daily activities and limiting or preventing them. Light sensation of dizziness, namely, if occurring without nausea, is not considered as a criteria for VM; this is considered to avoid dubious manifestations among migraine sufferers (see “ICHD-3 Beta Diagnostic Criteria of Vestibular Migraine”). Associated symptoms may occur before, during or after vestibular symptoms [3]. Different symptoms may occur in different episodes, but only one symptom is necessary during a single episode. Photophobia is very typical of migraine attacks but unusual in peripheral vertigo, thus contributing to increasing the specificity of diagnosis. Phonophobia is defined as sound-induced discomfort. It is usually bilateral and transient.

#### 3.4.2. Epidemiology

Recent epidemiological studies using the new diagnostic criteria are missing. Studies in tertiary headache clinics reveal great variability in prevalence in different series and according to the clinical context: 4.7–29.3% in an otolaryngology clinic and 9–11.9% in a headache clinic [7, 17, 26, 38]. A recently retrospective, observational, and descriptive study [40] performed in an outpatient clinic revealed that VM affects more middle-aged women than men and that migraine was diagnosed before vertigo in the majority of the situations [40].

In a large epidemiological study conducted in 2005, VM was shown to predominate in adult women, during the fourth-fifth decades of life, at a ratio of 1.5 to 5 : 1 [9]. Across life, the onset of vestibular symptoms arises generally 8 to 19 years after migraine with or without aura [18]. In postmenopausal women, episodes of vertigo, dizziness, or transitory imbalance without headache frequently occur [41, 42].

The relation between vertigo and migraine with or without aura is still epidemiologically controversial [43, 44]. The assumption of a more strong association between migraine without aura and vertigo [4, 26, 45] was recently questioned. A prospective and cross-sectional study of 462 consecutive patients who were present to consultation in a headache clinic and inquired about dizziness revealed that the prevalence of VM was higher (24.5% versus 12.1%) in migraine with aura, compared with migraine without aura [46]. When the intensity of migraine pain was classified as seven or greater in a Likert scale (out of 10), half of the subjects also reported vertigo [46].

Familial occurrence has been reported in some patients with an autosomal dominant pattern of inheritance and decreased penetrance in men. The genetics of VM are heterogeneous and uncertain, but several studies suggest linkage to chromosome 5q35 [47] or 22q12. A linkage to 22q12 in BPV, but not in migraine with aura, was found [48].

#### 3.4.3. Clinical Characteristics

According to the definition and diagnostic criteria, headache must be of moderate up to severe intensity in VM. However, nearly 30% of VM attacks never have associated headache. Only in a quarter or less of patients with throbbing migraine attacks, the crisis includes vertigo or dizziness and headache. On the edge, migraine and vertigo can never occur together. However, episodes should have other features that are typical of migraine (aura without headache, phono- and photophobia, nausea and vomiting aggravation with routine activities, etc.) [26, 49, 50]. Relief with sleep and a need to rest may help the diagnosis.

Patients frequently recognize trigger factors of migraine. Restriction or excess of sleep, stress, hunger or fasting, some type of food (cheese, red wine, aspartame or monosodium glutamate, chocolate, and strawberries), environmental factors (hot weather or changes of weather, altitude, and barometric pressure), physical activity, sensory stimulus (bright lights, unusual smells and sounds), menstruation, visual motion, are examples of that. All of them may contribute to trigger typical migraine and VM, but this one appears to have a reduced threshold to other triggers, such as Valsalva maneuver and head movements, for example.

#### 3.4.4. Vestibular Symptoms

Tinnitus, ear fullness, and hearing loss are reported by about 48% of migraine patients, causing difficulty in the differential diagnosis [14, 51]. However, hearing loss has no or only minimal progression over time [52]. The absence of these auditory symptoms supports the diagnosis of VM.

Vertigo of VM may be central or peripheral; therefore, this is not a defining feature of VM. A predominance of central vertigo seems to exist (50% versus 15% for peripheral vertigo), although in 35% the results are unclear, making this issue controversial [18]. Vertigo or dizziness may also be classified as spontaneous or as provoked [53]. Positional vertigo occurs in 40–70% of patients, not necessarily in every single attack [54, 55], with positional nystagmus present in 70–80% of patients [56]. It is generally a direction-changing horizontal nystagmus, sometimes appearing only with fixation suppression. Spontaneous nystagmus is rarer [52, 57].

The combination of numerous types of vertigo in vestibular migraine distinguishes it from other neurootologic disorders whose vertigo is monosymptomatic. When vertigo is positional it requires other data for analysis: in VM nystagmus does not occur in every crisis; when it appears, it may remain with the maintaining of the head posture and the episodes may last from minutes to days. The patient becomes hypersensitive to head movement and particularly to visual-dependent stimuli. Even between attacks visual-vestibular sensitivity and motion sickness are very frequent. More importantly though, positional evoked nystagmus cannot be explained by semicircular canal activation, in VM. Also, its resolution with liberatory maneuvers favors a diagnosis of benign paroxysmal positional vertigo (BPPV) as a nystagmus associated with a central cause should not resolve with physical maneuvers [58]. Although physical examination and nystagmus patterns may be of vital importance for VM differential diagnosis, they are not taken into account in the ICHD-3 beta diagnostic criteria for VM.

Physical examination is usually normal between attacks [45, 52]. In attack-free periods, spontaneous nystagmus is rare. Positional nystagmus of central origin occurs in 10–20% of patients [52, 56] and gaze-evoked nystagmus may be rarer [18, 52, 59]. However, a follow-up study by Radtke et al. has revealed oculomotor abnormalities between attacks in patients with VM of 16% initially up to 41% after 9 years [52], as corroborated by several other studies [26, 54]. The most frequent abnormalities are persistent positional nystagmus and saccades [26, 52], suggesting a delay in the recovery of vestibular dysfunction [52].

Vestibular dysfunction between attacks, evidenced by physiological tests, are not specific indicators of vestibular migraine per se [2, 9, 45] but may show signs of central and/or peripheral impairment. Although abnormal canal parameters are not characteristic for VM, the most consistent finding may be the unilateral weakness in caloric response [56], which affects 10 to 20% of patients with vestibular migraine [45, 54]. A bilateral caloric weakness further occurs in 11% of patients [56]. The head impulse test, also a test of canal function, may show abnormalities in more than 26% of patients [54, 60]; despite its quantification, the video head impulse test (vHIT) may be only abnormal in 11 to 15% of the patients [61, 62]. Vestibular evoked myogenic potentials may show decreased amplitudes either unilaterally or bilaterally [18, 63, 64]. Postural instability is usual in patients with VM [65], but posturography is considered normal in 74% of cases [54]. Pure-tone audiometry shows sensorineural impairment in only 7.5% of patients [66].

## 4. Differential Diagnosis

VM must be differentiated from other paroxysmal disorders associated with vertigo that may cooccur with migraine headaches.

### 4.1. Other Vestibular Disorders and Migraine

By definition, Ménière's disease (MD) is a chronic progressive disorder characterized by two or more recurrent episodes of spontaneous vertigo, lasting from 20 minutes to 12 hours and transient low to medium frequency sensorineural hearing loss in the affected ear before, during, or after one of the vertigo episodes on at least one occasion, as well as ipsilateral fluctuating aural symptoms (ear fullness or tinnitus). Other causes must be excluded. The major difference distinguishing definite from probable MD is the audiometric documentation of the hearing loss [67]. As with ICHD-3 beta diagnostic criteria of VM, MD classification does take into account vestibular or oculomotor examination.

Recently and according to Ghavami et al., the majority of patients with MD have migraine as defined by the International Headache Society and Bárány Society [68]. The increase in endolymphatic pressure is accepted as the cause of this disorder [69, 70]. Sensitivity to visual motion, light and sound, head motion, smells, weather changes, or medication was present in 95% of all patients with definite MD [71] (and also in migraine) patients. Caloric stimulation may also trigger migraine attacks in migraine patients [72, 73].

It is not easy to distinguish between these entities (Table 1). The first description of this association was done by Ménière in 1861, who named it apoplectiform cerebral congestion [74]. There is an impression of a continuous spectrum with different phenotypes regarding symptom intensity [68]. The correct diagnosis is important for treatment and prognosis. In most cases, the disease evolution through time allows the differential diagnosis, namely, with VM. Initially the symptoms can be very similar, but the progressive hearing loss in MD often progresses with or without vertigo. MD also predisposes to BPPV [75], eventually from inner ear injury with detachment of otoconia [71, 76]. Also, migraine is 3 times more common in patients with idiopathic BPPV than in BPPV secondary to trauma [71].

### 4.2. Anxiety Disorders, Migraine, and Vertigo

The term vertigo was used in the past to denominate three different disorders, currently recognized as vertigo, migraine, and panic disorder. Anxiety is a comorbidity factor with VM and MD [77]. Somatoform vertigo is also frequent in adults and children [61, 78, 79]. Patients with VM and MD exhibit high levels of psychopathology (65% and 57%, resp.) while in BPPV they were comparable to those present in the population without vestibular conditions [78].

### 4.3. Other Differential Diagnosis

The other events we need to think about are transient ischemia of the posterior circulation (transient ischaemic attack, TIA), vestibular paroxysmia (presumably caused by vascular compression of the vestibular nerve) [80], syncope, and orthostatic hypotension [4].

## 5. Pathophysiology of Vestibular Migraine

The exact mechanisms underlying the pathophysiology of vestibular migraine are still unclear and the majority of theories published so far mainly focus on the complex neural correlates of migraine, without clearly providing an explanation for vertigo. However, the interaction between the nociceptive and vestibular systems seems to be quite intuitive, since there is an evident and tight connection between the vestibular structures and brainstem areas related to the processing of pain such as dorsal raphe nuclei, the locus coeruleus, the lateral tegmentum, and connections between inferior, caudal, and lateral vestibular nucleus and the caudal trigeminal nucleus [45]. Thalamus afferent pathways of these nucleus are near and connected with common areas related to pain and vestibular cortex, insular cortex, orbitofrontal cortex, and cingulate gyrus [81] and strongly suggest the interaction and connection between both.

The evidence coming from studies and supporting the notion that the anatomical proximity of those structures justifies their intervention in the pathophysiology of VM is scarce, but a few experiments described a strong connection and increased signal transmission between vestibular nuclei and the nociceptive system, when comparing VM patients with controls. Some experiments using animal models also contributed to stress this relationship and to involve the activation of a trigeminovascular reflex within the inner ear, as a possible cause for local dysfunction in VM. In fact, in guinea pigs, the ophthalmic branch of the trigeminal nerve directly innerves the cochlear vessels and the stimulation of that nerve led to vasodilation in the inner ear with increased vascular permeability and plasma, protein, and 5-HT extravasation in that model [82]. In humans, by using a painful electrical stimulus on a skin area innervated by the trigeminal nerve (supraorbital point), but not in median nerve, in patients diagnosed with VM, a nystagmus could be induced or modified (increased), while in healthy controls nothing could be observed in the same experimental conditions [82–84]. These findings were interpreted as a signal of direct vestibular stimulation by the trigeminovascular system. Vestibular manifestations occur associated with cortical hyperexcitability and central sensitization of the trigeminal system, in migraine patients, due a specific impact over the vestibular system [82]. After that, several studies tried to replicate this experiment and corroborated the hyperexcitability of the vestibular system in migraine patients [85, 86]. Hyperexcitability of vestibulothalamocortical pathways is associated with activation of cerebellum and hypoactivity of occipital lobes, eventually suggestive of a reciprocal inhibition between vestibular and visual systems [81]. A decreased suppression of otoacoustic emissions was observed by Murdin et al., when comparing vestibular migraine patients with healthy controls [86]. Lewis et al. showed that the perceptual threshold of dynamic head movements was reduced in vestibular migraine patients, also when comparing with healthy controls [85]. The cause of this impairment of the vestibular system, presumed to result from cortical hyperexcitability, is not clear. Nevertheless, the theory of a “locus minoris resistentiae,” despite remaining unproven, seems to be plausible; the susceptibility results from diseases affecting the vestibular system (such as a neuritis) on the basis of a preexisting migraine.

Also the concept of “cortical spreading depression” has been invoked to explain the pathophysiology of vertigo in vestibular migraine [49]. Nevertheless, it is quite unlikely to have a cortical spreading depression on brainstem (and cortex) without causing any other sign or symptom. However, it is relevant to state that this notion is important for the differentiation of vestibular from basilar-type migraine, in which the headache should be accompanied by two different brainstem symptoms, at least. Neocortical and extracellular release of molecular signals during cortical spreading depression may contribute to the activation of trigeminal afferents on cranial blood vessels and this important step would elicit a trigeminovascular reflex-mediated vasodilatation in the meninges. This response would trigger a sterile inflammatory response extending also to the inner ear, as demonstrated in animal models [83, 84]. These local vascular changes may contribute to the perception of an abnormal balance, seeming to play a role in the pathophysiology of vestibular migraine.

Notwithstanding, vasodilatation is neither sufficient nor necessary for the perception of pain, and this is where the trigeminal afferent activation in ascending thalamocortical pathways comes into play [87]. These pathways comprise a group of structures and regions that current knowledge established as defining a central migraine circuit, which naturally includes central vestibular pathways. Data coming from human functional imaging studies show that, by stimulating the vestibular system, regions typically involved in the perception of migraine pain (such as the posterior and anterior insula, orbitofrontal cortex, and the cingulate gyri) are activated [81, 88]. In addition, trigeminal nociceptive and vestibular inputs are also received by the caudal parabrachial nucleus and this can contribute to motion hypersensitivity in migraine patients [89]. The parabrachial nucleus is in close relationship with the amygdala, in which there is a high expression of stress-response receptors. This suggests a role of stress in the development of migraine signs and symptoms [77] and also points to the existence of a cognitive-behavioral component in the disease and an interoceptive performance that seem to be modulated by the dorsal raphe nucleus and the locus coeruleus [45].

The relationship between migraine and episodic ataxia type 2, which is clearly associated with balance disturbances, raised the hypothesis of vestibular migraine to be a channelopathy [90]. In fact, mutations of the CACNA1A gene (which encodes a voltage-dependent calcium channel) were described both in episodic ataxia type 2 and in familial hemiplegic migraine, making plausible a genetic bridge between migraine and vestibular disorders [31]. However, so far, it was not possible to identify a consistent genetic defect involving CACNA1A gene in vestibular migraine patients [90]. It is known that the gene encodes a calcium channel that is predominantly expressed in the cerebellum and this fact may contribute to explain why familial hemiplegic migraine patients with CACNA1A mutations may frequently report central vestibular signs and symptoms. Notwithstanding, even in migraine with aura patients, not suffering from familial hemiplegic migraine and not having any kind of genetic defect involving CACNA1A, a dysfunction of calcium channels has been described, suggesting that this is a more generalized problem among patients with migraine [91].

In view of such complexity, not only in anatomical grounds, but also in terms of neurochemical organization of pain and vestibular pathways, it seems evident that more studies are needed to understand vestibular migraine pathophysiology and, furthermore, the features underlying the success of pharmacological intervention, where this aspect is critical for the design of successful clinical trials for vestibular migraine.

## 6. Treatment

So far, evidence for effective treatment in VM is scarce [92] and knowledge of therapeutic approaches is poor. Two randomized and controlled studies with triptans–*zolmitriptan* versus placebo during VM attacks and rizatriptan versus placebo in migraine patients, with or without dizziness in provoked motion sickness–produced inconclusive, because of the large confidence intervals and a small number of patients enrolled [93–95]. Authors suggest that serotonin agonists (5HT1A and 5HT1B) reduce vestibular-induced motion sickness by influencing serotoninergic vestibular-autonomic projections.

Despite the lack of empirical evidence, patients with VM are often treated with migraine prophylaxis, on the assumption that they may also control vestibular symptoms. Drugs such as calcium-channel blockers, namely, flunarizine and cinnarizine, are popular in VM prophylactic treatment. Cinnarizine was tested in a retrospective, single-centre, open-label study on VM and migraine associated with vertigo, in 1993, in 24 patients [37]. The conclusion was that the drug was safe and effective in reducing both headache and vertigo aspects of “migraine plus vertigo” among patients who suffer from either VM or MBA associated with vertigo [96]. A randomized controlled trial was performed in a tertiary centre with flunarizine versus beta-histine 16 mg and vestibular exercises. Frequency and intensity of vertigo decreased, but frequency and intensity of migraine were not significantly different between groups. A different retrospective study between flunarizine and propranolol showed that both were effective. But all the other preventive therapies have been used with efficacy: antiepileptic drugs (valproic acid, topiramate, and lamotrigine), beta-blockers (propranolol and metoprolol), and antidepressants (such as amitriptyline) magnesium and clonazepam [5]. A trial of onabotulinumtoxin type A (155 IU) revealed no significant benefit in improving VM [5]. Caffeine discontinuation and vestibular rehabilitation exercises were described as beneficial in patients with VM in addition to pharmacological treatment [5, 93, 97].

In the past, several studies relied on hardly reproducible aspects [93]. Patients identified according to different criteria were necessarily a heterogeneous group. Nevertheless, the success of preventive treatments suggested a similar pathogenesis in migraine and VM, but further studies are still missing [4].

## 7. Discussion/Conclusions

Considering that there are no specific and precise biomarkers, the differential diagnosis necessarily relies on clinical skills. Difficulties associated with symptoms and disease definitions, but also vestibular examinations, are immense. Most clinicians are not expert in vestibular semiology and the neurophysiological assessment of vestibular apparatus is not easily available. In addition, specialists observe the same pathology with different eyes. More neurologists believe that VM is a central problem rather than a peripheral one. Otolaryngologists see the opposite [45]. So, multidisciplinary intervention is crucial.

Given that it is fundamental to have a deep knowledge of the precise clinical features and natural history of each one of these entities, moreover, it is crucial to develop different tools that, in clinical grounds, may contribute to offer physicians the possibility of shaping treatments and changing prognosis. Naturally, the development of surrogate markers using new technological achievements is something that, in this field, has great avenues of research to go through. Functional and molecular imaging techniques are two of the tools that, certainly in the near future, will be helping to clarify many aspects of the complexity of the pathophysiology of these entities.

Genomics will help to personalize clinical intervention, not only in diagnostic terms, but also in facilitating therapeutic guidance and predicting treatment response and prognosis. These are only two examples of how the future might be promising in investigating these issues. It is therefore important to remain focused on bringing to clinical practice all the relevance of that scientific knowledge. The knowledge will allow a proper diagnosis and treatment and a consequently better quality of life of these patients.

## Figures and Tables

**Table 1:** Vestibular migraine, MD, and BPPV: distinctive features.

	VM	MD	BPPV
Age	Always (peak 4th decade)	3rd–5th decade	5th decade
Sex	F 3 : 1	F/M 1 : 1 or 1 : 2 M	F 2 : 1
Family history	++	+	+
Vertigo	Polysymptomatic, monosymptomatic, or positional	Monosymptomatic	Positional
Attacks duration	Minutes to days	20 minutes to 12 hours	Seconds, but recurrent positional paroxysms for days or weeks
Phono/photophobia	++	+	−
Headache	++	+	+−
Aura	+	−	−
Hearing loss	+	+++	−
Progressive hearing loss	−+	+++	−

## Endnotes

1. Code also for migraine diagnosis.

2. Vestibular symptoms (Barany Society's Classification of Vestibular Symptoms) qualifying for vestibular migraine include the following: (a) spontaneous vertigo: (i) internal vertigo (self-motion sensation) and (ii) external vertigo (spinning or flowing of visual surround); (b) positional vertigo, with head position change; (c) visually induced vertigo, occurring with visual stimuli of wide range; (d) head motion-induced vertigo; (e) head motion-induced dizziness with nausea (dizziness is assumed as a sensation of lack of spatial orientation).

3. Vestibular symptoms are considered moderate if they interfere with daily activities but do not prevent them or severe if they do not allow daily activities to be performed.

4. The episodes have variable duration, going from minutes to several days (or more, in a minimum number of subjects). Near 10% of patients have clusters of attacks lasting seconds. The total period of vertigo is defined as the total time of those episodes, even when they have short duration.

5. The symptoms can change from an episode to another and, in each episode, only one symptom is necessary. Other symptoms can be present in association with vertigo, during, previously, or after it.

6. Phonophobia is characterized as a bilateral and transient sound-induced discomfort, while recruitment is often a persistent unilateral discomfort.

7. Visual auras can be extremely variable, but the most frequent ones are scintillating and bright lights or spectral fortifications that expand gradually to the periphery of the eye field. Typically, they appear in 5–20 minutes and last less than one hour. Different types of aura (somatosensory, dysphasic, etc.) are more ambiguous in terms of diagnosis and they are not included in the criteria.

8. History and physical examination do not suggest another vestibular disorder or another disorder is ruled out by ancillary tests. Other disorders can be present as comorbid or independent conditions.
